# Photon Energy-Dependent Ultrafast Exciton Transfer in Chlorosomes of *Chlorobium tepidum* and the Role of Supramolecular Dynamics

**DOI:** 10.1021/acs.jpcb.3c05282

**Published:** 2023-08-23

**Authors:** Sean K. Frehan, Lolita Dsouza, Xinmeng Li, Vesna Eríc, Thomas L. C. Jansen, Guido Mul, Alfred R. Holzwarth, Francesco Buda, G. J. Agur Sevink, Huub J. M. de Groot, Annemarie Huijser

**Affiliations:** † MESA+ Institute for Nanotechnology, University of Twente, 7500 AE Enschede, The Netherlands; ‡ Leiden Institute of Chemistry, Leiden University, Einsteinweg 55, 2300 RA Leiden, The Netherlands; § Department of Chemistry and Hylleraas Centre for Quantum Molecular Sciences, University of Oslo, 0315 Oslo, Norway; ∥ Zernike Institute of Advanced Materials, University of Groningen, Nijenborgh 4, 9747 AG Groningen, The Netherlands; ⊥ Max Planck Institute for Chemical Energy Conversion, Stiftstraße 34-36, 45470 Mülheim an der Ruhr, Germany

## Abstract

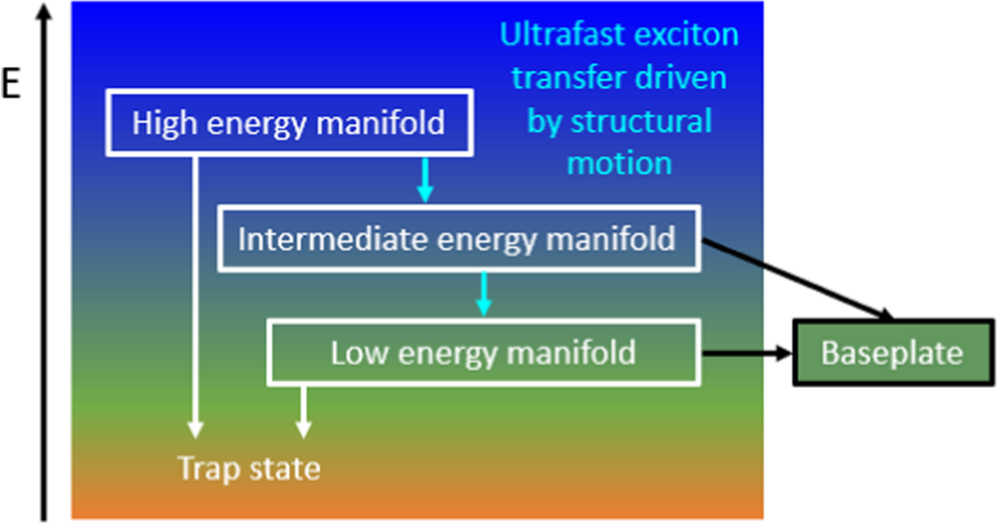

The antenna complex of green sulfur bacteria, the chlorosome, is one of the most efficient supramolecular systems for efficient long-range exciton transfer in nature. Femtosecond transient absorption experiments provide new insight into how vibrationally induced quantum overlap between exciton states supports highly efficient long-range exciton transfer in the chlorosome of *Chlorobium tepidum*. Our work shows that excitation energy is delocalized over the chlorosome in <1 ps at room temperature. The following exciton transfer to the baseplate occurs in ∼3 to 5 ps, in line with earlier work also performed at room temperature, but significantly faster than at the cryogenic temperatures used in previous studies. This difference can be attributed to the increased vibrational motion at room temperature. We observe a so far unknown impact of the excitation photon energy on the efficiency of this process. This dependency can be assigned to distinct optical domains due to structural disorder, combined with an exciton trapping channel competing with exciton transfer toward the baseplate. An oscillatory transient signal damped in <1 ps has the highest intensity in the case of the most efficient exciton transfer to the baseplate. These results agree well with an earlier computational finding of exciton transfer driven by low-frequency rotational motion of molecules in the chlorosome. Such an exciton transfer process belongs to the quantum coherent regime, for which the Förster theory for intermolecular exciton transfer does not apply. Our work hence strongly indicates that structural flexibility is important for efficient long-range exciton transfer in chlorosomes.

## Introduction

Green sulfur bacteria are anaerobic photosynthetic bacteria capable of sustaining life at extremely low-light conditions. Their habitats include microbial mats in hot springs and deep water regions down to 100 m in the Black Sea.^1^ What enables the survival of these bacteria in extremely low photon flux is their highly efficient light-harvesting antenna complex, the chlorosome, which unlike most photosynthetic light-harvesting complexes does not rely on a protein scaffolding.^2,3^ This makes the chlorosome of particular interest for applying its design principles to artificial antenna systems. A single chlorosome consists of up to 250.000 self-assembled bacteriochlorophyll (BChl) *c*, *d*, or *e* molecules, which along with carotenoids and quinones form a multitubular supramolecular structure.^4^ The size of the chlorosome is typically in the range of 100–200 nm in length and 40–60 nm in diameter, depending on the species and the light intensity during growth.^5,6^ Encased within a lipid-like envelope layer, the multitubular structure is attached to the interior of the cytoplasmic membrane via a pigment–protein complex containing BChl *a* molecules known as the baseplate.^5^ The baseplate is anchored to the cytoplasmic membrane where a type 1 reaction center is embedded with the Fenna–Matthews–Olson protein (FMO) complex based on BChl *a* molecules, carotenoids, and proteins.^7,8^ The unique ability of green sulfur bacteria to live under such extremely low-light conditions is made possible by the highly efficient transport of excitons (bound electron–hole pairs) generated by light absorption, through the chlorosome toward the baseplate and the reaction center, where the primary charge separation step occurs.^5^

To understand how the chlorosome antennae ensure such highly efficient long-range exciton transport, various fs spectroscopy studies have investigated the exciton transfer processes and dynamics. Earlier works report that following photoexcitation, exciton relaxation within individual tubes occurs in 100–300 fs, followed by intertube exciton transfer in a few ps and exciton transfer to the baseplate in ∼10 to 140 ps and to the FMO complex and reaction center in ∼200 to 300 ps.^9−17^ However, due to spectral overlap in various transient signals, the assignment of time constants is inherently ambiguous and the exciton transfer mechanism is still discussed.^17−20^ Moreover, it has been demonstrated that photon densities higher than ca. 1.5 × 10^12^ photons/cm^2^/pulse result in significant exciton–exciton annihilation,^21^ distorting the exciton transfer dynamics and behavior that largely deviates from the native environment. In contrast to these timescales from experimental studies, recent combined quantum chemical and molecular dynamics (MD) studies indicate exciton delocalization over the entire chlorosome tubular structure to occur much faster, even less than 1 ps.^19,22^

In addition to its highly efficient exciton transport toward the baseplate, a common feature observed for chlorosomes in femtosecond transient absorption (TA) and 2D electronic spectroscopy experiments is a number of prominent short-lived oscillations. In particular, the species *Chlorobium tepidum* shows two characteristic frequencies of 70–90 and 130–160 cm^–1^.^9,10,23^ It is unknown whether these features, which possibly have a vibrational origin, are related to the highly efficient long-range exciton transfer.^17,19,22−24^ Understanding the exciton mechanism and in particular the relationship between structural motifs and function is both important from a fundamental perspective and to develop new design strategies for artificial antenna systems.

We propose that coupling of excitons to thermal motion plays an important role in the efficient long-range exciton transfer process in chlorosomes. This hypothesis is supported by our recent quantum-classical Frenkel exciton treatment based on MD simulations carried out at room temperature, showing that dynamic disorder promotes exciton transport by vibrationally induced recurrent transient nonadiabatic coupling of exciton states. In this work, we found that dynamic disorder promotes level crossings between exciton states, thereby promoting long-distance excitation energy transfer between domains of high exciton population.^19^ In our earlier work on exciton dynamics in layers of self-assembled porphyrin derivatives, we distinguished between (1) exciton delocalization and transport via a band mechanism and (2) exciton transport driven by thermal motion so that the exciton energies at the initial and final molecular site become temporally equal and energy transfer can take place.^25^ We postulate that the 1st mechanism mainly applies to low temperatures or highly structured systems, for example, crystals of naphthalene or anthracene molecules,^26,27^ and the 2nd mechanism to supramolecular systems like the chlorosome with some static and dynamic structural disorder.^5,28−30^ The latter mechanism is possible in case the energy difference involved in dynamic structural disorder is similar or close to the energy gap between exciton states.^31,32^ We postulate that vibrationally induced quantum overlap between exciton states promotes the highly efficient exciton transport in the chlorosome of *C. tepidum*.^19,33,34^

The present broadband femtosecond TA study aims to shed light on these questions. Our work indicates that excitation energy is distributed very rapidly, and is delocalized over the chlorosome in <1 ps. We observe a so far unknown impact of the excitation photon energy on exciton transfer in the chlorosome of *C. tepidum* at room temperature. This demonstrates the presence of distinct optical domains where one is favored over the other for exciton transfer toward the baseplate, FMO complex, and the reaction center. The most efficient exciton transfer appears to be correlated with the most intense short-lived oscillatory feature that is observed, suggesting a correlation. Our work hence strongly indicates that structural flexibility is important for long-range exciton transfer in self-assembled supramolecular structures with static and dynamic structural disorder like the chlorosome.

## Experimental Methods

### Sample Preparation

*C. tepidum* was grown anaerobically in a growth chamber at 40 °C using Wahlund medium in 1 L reactor bottles with continuous stirring and illumination with fluorescent tubes (Osram, mixture of 18W/25 universal white and 18W/77 Floura) using high light conditions at the surface of the bottles, and cells were isolated following the procedure in earlier work^35^ after 2 days of growth. For all measurements performed, *C. tepidum* samples were diluted with 50 mM Tris-HCl buffer solution (Sigma-Aldrich, pH = 8.0), which was deaerated by bubbling N_2_ through the solution for 1 h, to an absorbance of around 0.3 optical density (OD) at the BChl *c* Q_y_ wavelength of maximum absorption λ_max_ = 751 nm in a flow-through cell (Hellma, 1 mm optical path length, flow rate of 20 cm/s). In addition, 5 mM sodium dithionite (Sigma-Aldrich) was added to the deaerated buffer to achieve anaerobic conditions and avoid O_2_-induced quenching,^34^ and the suspension was incubated in the dark for 2 h in an airtight vessel.^17^ The flow-through cell was mounted on a home-built motorized stage moving with a rate of ca. 1 mm/s perpendicular to the flow direction to regularly refresh the sample and avoid photoinduced damage, which was verified by identical UV–vis spectra of the samples before and after the experiments. All experiments were performed at room temperature (20 ± 1 °C).

### UV–Vis Absorption Spectroscopy

The UV–vis spectra were measured using a Shimadzu 1800 UV–vis spectrometer, with the sample contained in a 1 mm optical path length micro cuvette (Hellma, absorption cuvette).

### Transient Absorption Spectroscopy

The fs TA measurements were performed with a home-built pump-probe setup. Seed pulses from a Ti:Sapphire oscillator (Micra, Coherent) were amplified to 3.7–3.8 W with a Ti:Sapphire regenerative amplifier (Legend Elite, Coherent) equipped with a Nd:YLF Q-switched pump laser (Revolution, Coherent) to produce an 800 nm pulse with 35 ± 1 fs full width at half-maximum (FWHM) at 5.0 kHz repetition rate. The output was split into two paths using an 85:15 beam splitter to produce the pump and probe pulses. The major part of the beam was sent into an optical parametric amplifier (OPerA Solo, Coherent) to generate a pump pulse with tunable wavelength for excitation, which was then guided through a prism pair (LakL21, Edmund Optics) to compress the pulse to a near Fourier-transform-limited pulse with a center wavelength of 730, 740 or 750 nm (FWHM = 21 ± 1 nm) with 38 ± 1 fs FWHM pulse duration and focused on the sample with a diameter of ∼250 μm. A chopper wheel was used to reduce the pump frequency to 2.5 kHz, corresponding with half the repetition rate of 5.0 kHz, to allow sequential detection of pump-on and pump-off signals and determine the differential absorbance Δ*A*. The pump intensity was set at 1.0 ± 0.1 × 10^12^ photons/cm^2^/pulse to avoid exciton–exciton annihilation^21^ perturbing the dynamics at higher excitation intensities (Figure S1). To produce the white light probe, the remaining portion of the 800 nm fundamental was attenuated and focused into a 3 mm CaF_2_ window (Newlight Photonics, 001-cut) mounted on a continuously moving translational stage to avoid heating of the crystal and yielding a stable continuum from ca. 350 to 825 nm. After attenuation of the remaining fundamental using an 800 nm notch filter (FWHM = 40 nm) and a NENIR30B filter from Thorlabs, focused at the sample position with a diameter of ca. 100 μm to ensure the signal was probed on a homogeneously excited sample. The probe pulse was delayed with respect to the pump pulse using a mechanical delay stage. The white light probe pulse was detected by a 15 cm spectrograph coupled to a 256 segment photodiode array. The polarizations of pump and probe beams were set at the magic angle (54.7°) relative to each other.^36^ The instrumental response time (IRT) at 730, 740, or 750 nm photoexcitation, as determined from measuring the sum frequency of the pump and probe at the sample position in a 25 μm thick BBO crystal, equals ca. 80 fs. Although the continuum is stable, strong light absorption implies that less probe photons reach the detector, lowering the signal-to-noise ratio. As a result, the signal-to-noise ratio depends on the probe wavelength and is the best in case of weak absorption by the sample. The estimation and correction for the chirp of the transient absorption data was carried out using Matlab. Target analysis of the TA data was performed using the open-source program Glotaran.^37^

## Results and Discussion

**Figure 1:**
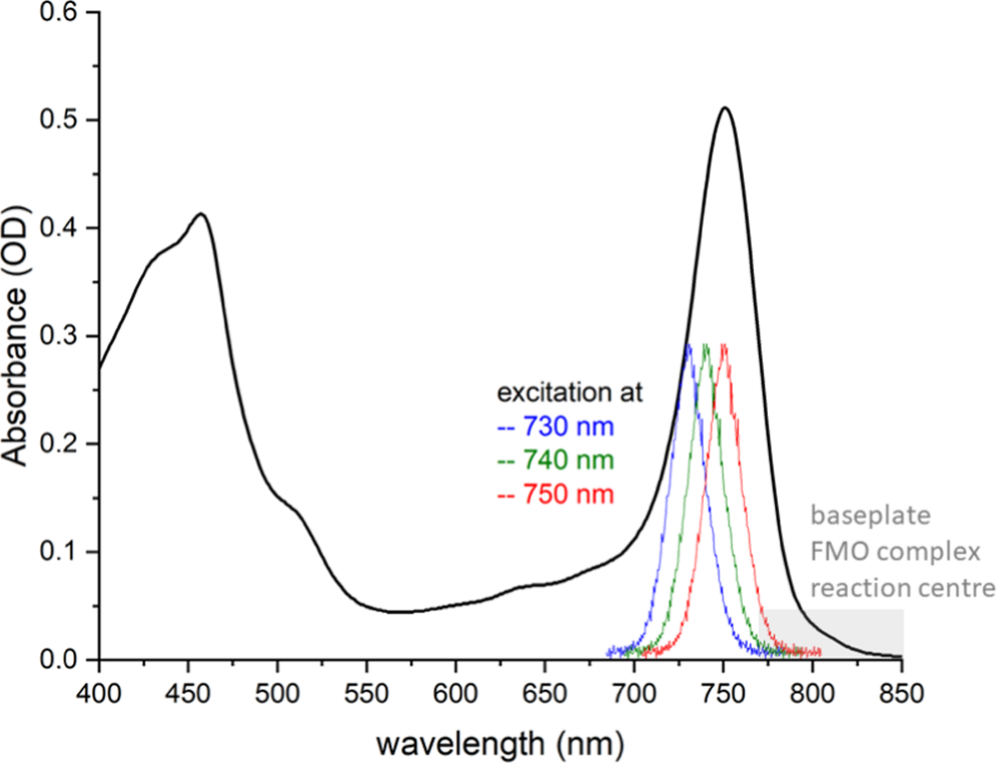
UV–vis spectrum of *C. tepidum* in deaerated 50 mM pH 8.0 Tris-HCl buffer at room temperature, normalized to the maximum of the Q_y_ band at 751 nm, and the profiles for excitation at 730, 740, and 750 nm (FWHM = 21 ± 1 nm, 38 ± 1 fs pulse).

The absorbance spectra of BChl molecules are composed of four bands, namely, the low-energy Q_y_ and Q_x_ bands and the high-energy B_y_ and B_x_ bands.^38^ The steady-state absorbance spectrum of purified chlorosomes of *C. tepidum* is shown in Figure 1. The absorbance maxima of the BChl *c* Soret band and Q_y_ band are observed at 457 and 751 nm, respectively, while carotenoids also contribute to the absorption in the blue-green region. The spectrum is red-shifted and broadened in comparison to monomeric BChl *c* in ethanol.^39^ The red shift is a consequence of strong exciton coupling, leading to exciton delocalization.^18,40^ Since strong exciton delocalization causes exchange narrowing, the spectral broadening is a consequence of the gross structural disorder in the chlorosome self-assembly.^41^ Hole burning studies have shown it is not possible to burn holes in the vicinity of the chlorosome absorption band maximum, indicating a major role of homogeneous spectral broadening.^42^ Especially above ca. 770 nm, the baseplate, FMO complex, and reaction center are also weakly absorbing.^43,44^

**Figure 2:**
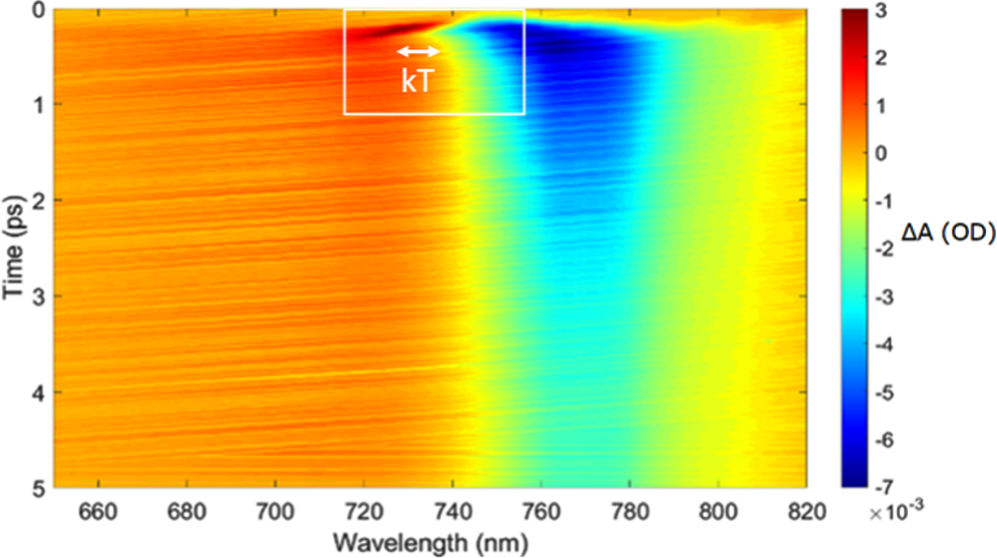
Two-dimensional TA map of *C. tepidum* in deaerated 50 mM pH 8.0 tris-HCl buffer at room temperature, showing the differential absorbance following 740 nm photoexcitation predominantly exciting the chlorosome. Note the strongly damped oscillatory feature (white box) with a shift in wavelength approximately corresponding to *k*_B_*T*.

**Figure 3:**
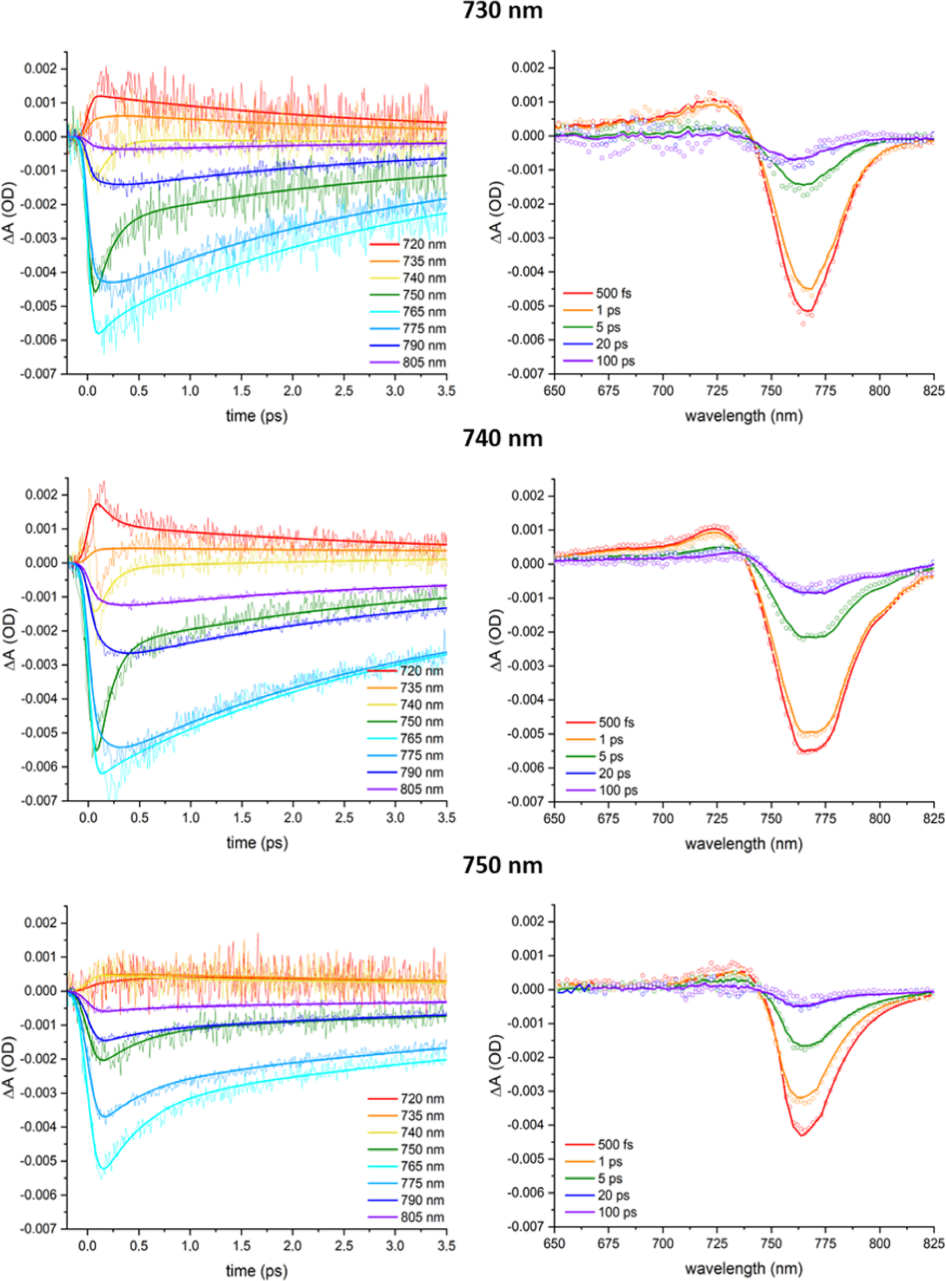
TA data (scatter) and fits from target analysis (solid lines) of *C. tepidum* in deaerated 50 mM pH 8.0 tris-HCl buffer at room temperature, obtained by excitation at 730, 740, or 750 nm. (Left) Kinetic traces at the indicated wavelengths; (right) spectra at various time delays.

The excitation energy transfer processes in the chlorosome of *C. tepidum* have been studied using fs TA spectroscopy, by exciting the blue side of the Q_y_ band at 730, 740, or 750 nm to preferentially excite the chlorosomes (Figure 1). The excitation intensity has been set at 1.0 ± 0.1 × 10^12^ photons/cm^2^/pulse, such that exciton–exciton annihilation^21^ is avoided and the decay normalized to a maximum signal intensity of −1 is independent of the excitation intensity (Figure S1). Figure 2 shows the TA contour plot of the early time kinetics for 740 nm excitation, plots for 730 and 750 nm excitation are provided in Figure S2. The positive TA signal from ca. 650 to 740 nm is due to excited state absorption (ESA). The broad negative TA signal in the range of ca. 740–820 nm arises from the ground state bleach (GSB) and stimulated emission (SE).^11^ Note the short-lived strongly damped oscillatory-type transient signal around 730–750 nm, i.e., in the region where the positive ESA signal has approximately the same intensity as the negative GSB and SE signals. The signal probed at e.g., 735 nm is first positive, then negative, weakly positive again and then decaying (see Figure 3). As the pump spectrum is also in this region, the assignment of this transient signal is complicated. The oscillatory signal is also observed following 730 or 750 nm excitation (see Figure S2), albeit with lower intensity than with 740 nm excitation, and especially with 750 nm excitation it is very weak, indicating that nonlinear optical processes are of minor importance. Instead, we attribute the feature to the chlorosome, and it suggests a different oscillatory behavior in time of the ESA vs the GSB and SE signals, i.e., different dephasing dynamics for excited state and ground state coherences. Low-frequency vibrational modes are well known for chlorosomes^10,20,23,45−48^ and artificial mimics.^49^ For *J*-aggregates such oscillatory behavior was assigned to coupling of excitons to phonons, changing the exciton delocalization length in time.^50^ Our recent MD, quantum chemical, and response function calculations on a chlorosome model structure indicate exciton delocalization over tens to hundreds of molecules.^18^ The shift in wavelength with time following photoexcitation equals ca. 10 nm, which is in the order of the thermal energy *k*_B_*T* (Figure 2, inset). The strongly damped oscillation has a main period of ca. 251 fs, which corresponds to a wavenumber of ca. 133 cm^–1^. Furthermore, Fourier transform of the residual of experimental and modeled data (Figure S3) reveal contributions around 91 and 66 cm^–1^. These frequencies are quite close to the 138, 89, and 57 cm^–1^ vibrational modes observed by resonance Raman studies,^46^ indicating a vibrational or mixed electronic-vibrational rather than a purely electronic origin, as detailed below. This interpretation is in line with earlier 2D electronic spectroscopy studies reporting 91 and 145 cm^–1^ coherent beatings dephasing in ca. 1.2 ps.^23^ Furthermore, the build-up time of the negative signal depends on the probe wavelength (Figure S4), indicating downhill exciton transfer.

Figure 3 compares selected time traces for excitation at 730, 740, and 750 nm (left panel) along with the TA spectra at varying delay times (right panel). The positive ESA signal from ∼650 to 735 nm develops within the instrumental response time (IRT, ∼80 fs) and then decays. The negative GSB and SE band above ca. 735 nm, with the GSB/SE ratio depending on the photoexcitation wavelength (Figure S5), clearly shows distinct spectral regimes. The signals ≤750 nm develop within the IRT, followed by a ps decay. In contrast, the build-up ≥750 nm occurs slower than ≤750 nm, demonstrating exciton transfer from higher to lower energy levels in the chlorosome exciton manifold, and explaining why the decay is slower at lower energies. Above ca. 780 nm the dynamics normalized to a maximum signal intensity of −1 are independent of the probe wavelength (Figure S6). Analogous to earlier work,^12^ we therefore assign this spectral range to arrival of excitons at the lowest states in the chlorosome exciton manifold and the baseplate. A weak negative signal is persistent at later times, indicating that subsequent exciton transfer from the baseplate to the FMO complex and reaction center occurs on a sub-ns timescale.^11^

**Figure 4:**
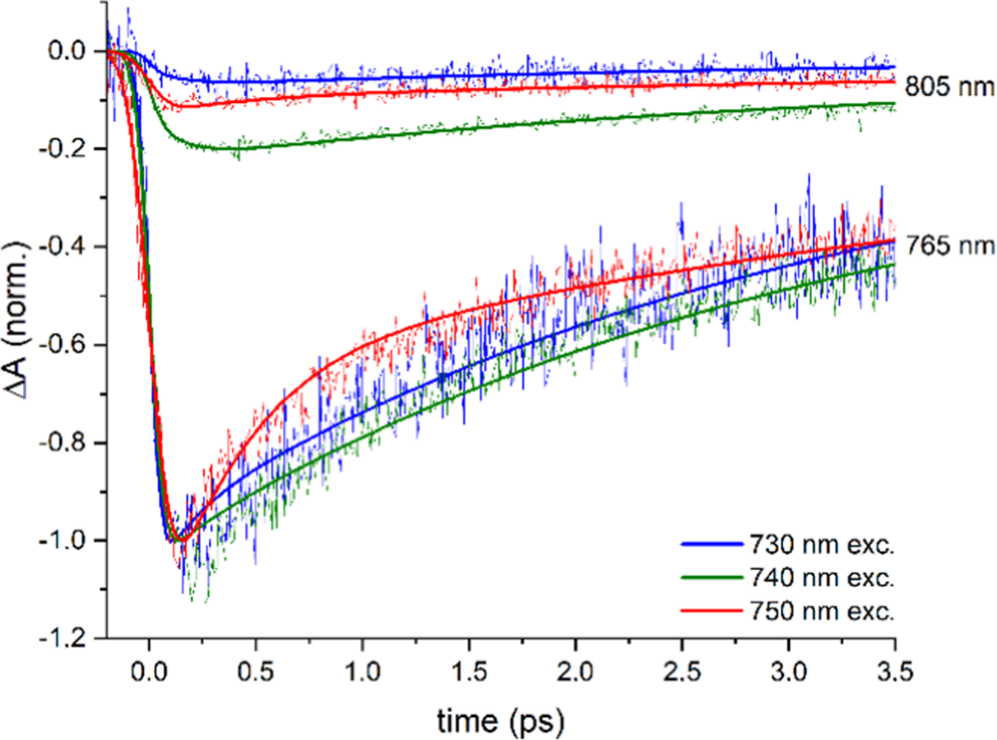
Kinetic traces at 805 nm (scatter) and fits from target analysis (solid lines) of *C. tepidum* in deaerated 50 mM pH 8.0 tris-HCl buffer at room temperature for 730, 740, and 750 nm excitation, normalized to the maximum intensity of the transient signals at 765 nm also shown.

Figure 4 shows the kinetic traces probed at 765 and 805 nm for excitation at 730, 740, and 750 nm, normalized to the minimum intensity of the TA signal at 765 nm (Figure 3). The decay at 765 nm only slightly depends on the excitation wavelength (Figure S5b), indicating an almost similar GSB/SE ratio for excitation at 730, 740, and 750 nm, in contrast to e.g., the kinetic trace at 750 nm (Figure S5a), causing the latter to be unsuitable for normalization. The raise of the signal at 805 nm, assigned to arrival of excitons at the lowest exciton manifold of the chlorosome and the baseplate, appears to be slightly slower especially for 730 nm excitation compared to excitation at 740 or 750 nm, although the signal is very weak (Figure S7), followed by a similar decay for excitation at 730, 740 and 750 nm. The signal at 805 nm is the most intense for excitation at 740 nm (defined as 100%), indicating the most efficient exciton transport to the baseplate, and substantially less intense for excitation at 730 nm (∼30%) or 750 nm (∼55%). Interestingly, the most efficient exciton transfer observed at 740 nm excitation coincides with the most intense oscillatory feature, suggesting a correlation. This is supported by the similar <1 ps time windows in which dampening of the oscillatory signal and downhill exciton transfer in the chlorosome exciton manifold occur. The higher exciton transfer efficiency at 740 nm relative to 730 or 750 nm excitation indicates the presence of distinct optical domains, in agreement with single-chlorosome studies,^28,51,52^ and the competition between downhill exciton transfer through the chlorosome toward the baseplate with a loss channel in the chlorosome, possibly to a low-energy charge transfer state.^53^

**Figure 5:**
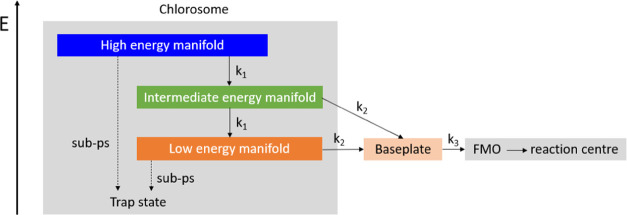
Photophysical model used for target analysis; blue, green, and dark and light orange boxes are assumed to contribute to the TA signal. The absorption of the baseplate is expected to spectrally overlap with the low-energy manifold of the chlorosome, but with weaker absorption. The dashed arrows indicate exciton trapping to account for the excitation wavelength dependency observed.

A sequential photophysical model with three components can describe the TA data except the oscillatory feature but does not explain the impact of the excitation wavelength observed here and has therefore not been used further. Instead, the observed trends are well explained by the model shown in Figure 5 and the time constants presented in Table 1, as clear from the fits included in Figures 3 and 4. The species-associated spectra of the optical components of this model are presented in Figure S8. Earlier hole burning studies reported at least two spectrally distinct domains in the chlorosome of *C. tepidum*: (1) higher excitonic states with the main oscillator strength and a maximum absorption around 750 nm and (2) the lowest excited states of the BChl *c* aggregates absorbing from ca. 760 to 800 nm.^11^ Our model is based on three exciton manifolds in the chlorosome: with high energy, intermediate energy, and low energy. Especially for *C. tepidum* the lowest-energy manifold of the chlorosome spectrally overlaps with the weaker baseplate absorbance,^12^ which can explain why ≥780 nm the kinetic traces normalized to a differential absorbance of −1 are independent of the probe wavelength (Figure S6). The weak contributions to the absorbance of the FMO complex and reaction center (Figure 1) have not been included in the model. More than three exciton manifolds may exist in the chlorosome; single-chlorosome studies indicate a high-energy doublet and a low-energy doublet.^28,51^ However, as these doublets have spectral overlap and the model based on three exciton manifolds in the chlorosome describes the TA data well, we decided to restrict ourselves to the most basic model possible. In addition, the model is motivated by the observation that the downhill exciton transfer is the most efficient at 740 nm excitation, and less efficient for excitation at 730 or 750 nm. This indicates an exciton trapping channel, with the strongest impact at 730 or 750 nm excitation, and possibly involving a charge transfer state,^53^ that is competing with downhill energy transfer with rate constant *k*_1_ through the chlorosome exciton manifold toward the baseplate. According to this model, a decrease in excitation photon energy implies less excitation of the high-energy manifold and more excitation of the low-energy manifold or baseplate. The time constants do not need to depend on the excitation wavelength, and indeed only small differences are observed for excitation at 730, 740, and 750 nm (Table 1). Only for the latter 1/*k*_1_ and 1/*k*_2_ are slightly longer, which may be due to photoexcitation relatively low in the exciton manifold of the chlorosome. Hole burning studies have shown it is not possible to burn holes in the vicinity of the chlorosome absorption band maximum,^42^ indicating homogeneous spectral broadening. If not trapped into a charge transfer state, an exciton becomes delocalized over the chlorosome in <1 ps, which is in line with recent quantum chemical and MD studies^22^ in which we observed that dynamic disorder promotes exciton delocalization over the chlorosome on a timescale of less than 1 ps.^19^ An exciton can subsequently be transferred to the baseplate with rate constant *k*_2_, possibly via an incoherent Förster-type hopping mechanism,^54,55^ and from there on a (sub-)ns timescale^11^ to the FMO complex and reaction center with rate constant *k*_3_. The transfer time to the baseplate is in line with earlier work reporting a value of ∼12 ps for *C. tepidum* at room temperature,^12^ but significantly faster than the 30–40 ps for *C. tepidum* at 5 and 65 K,^11^ which can be explained by the thermal deactivation of coherent transfer and activation of Förster-type exciton transfer.^25^

**Table 1:** Time Constants Obtained from Target Analysis^a^

excitation center λ (nm)	1/*k*_1_ (fs)	1/*k*_2_ (ps)	1/*k*_3_ (ps)
730	115 ± 5	3.04 ± 0.02	∞
740	103 ± 3	3.21 ± 0.02	∞
750	330 ± 15	5.35 ± 0.05	∞

a The associated species-associated spectra are presented in Figure S8. The value of *k*_3_ has been set at ∞, i.e., longer than the experimental time window.

The impact of the excitation photon energy on exciton transfer in the chlorosome of *C. tepidum* at room temperature resolved in this study demonstrates optical domains where one is favored over the other for exciton transfer toward the baseplate. The most efficient exciton transfer at 740 nm excitation coincides with the most intense damped oscillatory feature observed. Our work hence contributes to converging evidence that structural flexibility is important for long-range exciton transfer in supramolecular structures with static and dynamic structural disorder like the chlorosome. The distinct optical domains can result from static and dynamic disorder at various levels. Structural inhomogeneity between chlorosomes as observed in single-chlorosome studies by Köhler and colleagues^28,51^ reporting high-energy and low-energy doublets likely plays a role. Each energy doublet is assigned to a pair of superradiant states with transition dipoles parallel and perpendicular to the tubular structure.^28,51,52^ Superradiance likely results from space-time averaging of exciton density over an ensemble of states of the cylindrical suprastructure.^18^ The 182 ± 45 cm^–1^ average energy spacing between exciton levels 2 and 3 in the work of Köhler^51^ may lead to the ∼133 cm^–1^ oscillatory signal. Other exciton levels interact as well and may give rise to the minor contributions around 91 and 66 cm^–1^ identified in this study (Figure S3). Also the variety in diameters of the tubular structures may contribute to static structural disorder and inhomogeneous spectral broadening since the inner rolls with the smallest diameter have a blue-shifted absorption compared to outer tubes.^12,28,56,57^ The nature of a BChl *c* molecule as a H-bond donor or not can affect the angle between molecules^18^ and as a consequence exciton delocalization. At the molecular level, heterogeneity in the 8 and 12 side chains for wild-type *C. tepidum* also contributes to static structural disorder.^28^ Spectral broadening could furthermore be due to coupling of excitonic states to charge transfer states, typical for closely packed molecules and reported for chlorosomes.^58^ The loss channel competing with exciton transfer (Figure 5) may be due to exciton trapping at low-lying charge transfer states known for excitonically coupled BChl molecules.^53^ Such trapping process can explain why the intensity of the TA signal above ca. 780 nm relative to that of the GSB (Figure 4) depends on the excitation wavelength, as it competes with the functional energy transfer processes described by *k*_1_ and *k*_2_ toward the baseplate.

The main feature at 133 cm^–1^ and weaker contributions around 91 and 66 cm^–1^ (Figure S3) are quite close to the 138, 89, and 57 cm^–1^ vibrational modes observed by resonance Raman studies,^46^ indicating a vibrational or mixed electronic-vibrational rather than a purely electronic origin. The oscillation may arise from interference between closely spaced exciton levels induced by structural disorder,^59^ which is supported by the width of the spectral fluctuation (white box Figure 2) approximately corresponding to an energy fluctuation of *k*_B_*T*. Gillbro et al. assigned low-frequency oscillations observed for chlorosomes of *Chlorobium phaeobacteroides* at room temperature to coherent ground state vibrations, with the vibrational coherence induced by resonant impulsive Raman scattering. An important difference with the present work is that their oscillations are longer-lived, which allowed us to observe 4–5 cycles before damping in ca. 1.5 ps,^47^ while the only 1–2 cycles in the present work suggest a different cause. Further indications for an excited state origin rather than a ground state Raman feature are the dependency on the excitation wavelength (Figure S2). Our MD simulations show that the oscillatory feature could originate from the rotational degree of freedom along the Mg–OH coordination bond.^18,29^ Such rotational motion in a cage is the signature of a plastic crystal, in which self-assembled molecules have an orientational degree of freedom within a crystalline network.^33^ Rather than by a monomeric vibrational origin, the dominant ca. 133 cm^–1^ feature here can hence be explained by a cooperative effect: a fluctuation in relative angle between the BChl *c* molecules in time.^18,29^

A key question is whether the strongly damped oscillatory feature observed is related to exciton transport. Whether structural dynamics play a vital role in efficient long-range exciton transfer is a highly debated topic and yet to be elucidated.^19,22−24^ Note that quantum coherent effects can also occur under incoherent illumination conditions and do not require a coherent light source.^60^ The strongly damped oscillatory feature is the most intense at 740 nm excitation and coincides with the most efficient exciton transport to the baseplate, suggesting a correlation. This is supported by the photophysical modeling (Figure 5), indicating that exciton transport through the chlorosome toward the baseplate occurs during the time the oscillation dampens. Also work by Chin,^32^ Aspuru-Guzik^22^ and our recent MD simulations^19^ show that dynamic disorder promotes exciton transfer in chlorosomes and leads to delocalization over the entire chlorosome in <1 ps. Such mechanism is possible in case the energy difference involved in dynamic disorder is similar or close to the energy gap between exciton states, which is defined as the intermediate-coupling regime where exciton transfer occurs the fastest.^31^ Such mechanism belongs to the coherent regime, for which the Förster theory for intermolecular exciton transfer^54,55^ does not apply. Instead, exciton diffusion can be described by a coherent part and an incoherent part.^25,61,62^ Structural dynamics disturb the coherent evolution (the incoherent contribution) and at the same time introduce new coherences (the coherent contribution).^19^ Based on these results, we propose that exciton transfer through the chlorosome is driven by structural motion. Analogous to earlier work on *J*-aggregates in which an excitation wavelength-dependent oscillatory signal was attributed to exciton delocalization,^50^ we assign the strongly damped oscillatory feature here to fast exciton delocalization (*k*_1_ in Figure 5). Exciton delocalization may occur over a single tube, several tubular structures, or over the entire chlorosome. Our quantum chemical and MD calculations on a chlorosome consisting of 3 nested tubes show that <1 ps exciton delocalization due to vibrationally induced transient nonadiabatic conversions occurs uniformly over this entire model chlorosome.^15^ The loss channel competing with exciton transfer may be due to exciton trapping at low-lying charge transfer states known for excitonically coupled BChl molecules.^53^ Although the fast delocalization implies a similar exciton density over the entire chlorosome, this process will cause the exciton to mostly reside on the outer tube due to the larger quantity of BChl *c* molecules, from where it can be transferred either back into inner tubes, or toward the baseplate (*k*_2_). Such a mechanism implies that in a self-assembled electronically coupled system with some static and dynamic structural disorder like the chlorosome, reducing structural flexibility moves the system away from the intermediate-coupling regime optimal for exciton transfer. In summary, the present work strongly indicates that low-frequency vibronic motions, likely due to rotation along the Mg–OH coordination bond,^18,29^ promotes long-range exciton transfer. The energy involved in this rotational motion is likely suitable for level crossing, and therefore selected instead of other vibrational modes.^63−65^ After the level crossing, the exciton will delocalize and couple to the multiple vibrational motions occurring in the chlorosome. This sheds light on a longstanding question on the role of molecular motions in long-range exciton transfer in chlorosomes, enabling new design strategies for artificial antenna systems.

## Conclusions

The present study aims at uncovering the mechanism of the highly efficient long-range exciton transfer in the chlorosome of *C. tepidum* and the role of quantum coherence, allowing green sulfur bacteria to survive in habitats with extremely low-light conditions. Our work shows that excitation energy is delocalized over the chlorosome <1 ps. The following exciton transfer to the baseplate occurs in ∼3 to 5 ps, in line with earlier studies performed at room temperature, but significantly faster than at cryogenic temperatures. Importantly, we observe a so far unknown impact of the excitation photon energy on exciton transfer, which demonstrates the presence of distinct optical domains where one is favored over the other for exciton transfer toward the baseplate. The most efficient exciton transfer coincides with the most intense damped oscillatory component observed, indicating a correlation. These results can be explained by exciton transfer promoted by low-frequency vibronic motions, likely originating from the rotation of molecules in the chlorosome. Such a mechanism belongs to the coherent regime, for which the Förster theory for energy transfer does not apply. The present study sheds light on a longstanding question on the importance of molecular motion for efficient long-range exciton transfer, enabling chlorosomes to be one of the most efficient supramolecular antenna systems in nature, and providing an ideal model system for the design of artificial systems.

## Supporting Information Available

Supplementary TA data, Fourier transforms of the residuals of experimental and modeled data, and species-associated spectra (PDF)
