# Supplementary material for: Photon Energy-Dependent Ultrafast Exciton Transfer in Chlorosomes of Chlorobium tepidum and the Role of Supramolecular Dynamics
Source: J Phys Chem B. 2023 Aug 23;127(35):7581–9. doi: 10.1021/acs.jpcb.3c05282 (PMC10493955; doi:10.1021/acs.jpcb.3c05282)
Supplement: Supplementary file 1 — jp3c05282_si_001.pdf [file jp3c05282_si_001.pdf]

Supporting Information

Photon Energy Dependent Ultrafast Exciton Transfer in Chlorosomes of Chlorobium Tepidum and the Role of Supramolecular Dynamics

Sean K. Frehan[1], Lolita Dsouza[2], Xinmeng Li[2,3], Vesna Eríc[4], Thomas L.C. Jansen[4], Guido Mul[1], Alfred R. Holzwarth[5], Francesco Buda[2], G. J. Agur Sevink[2], Huub J.M. de Groot[2] and Annemarie Huijser[1*]

[1]*MESA+ Institute for Nanotechnology, University of Twente, 7500 AE, the Netherlands*
[2]*Leiden Institute of Chemistry, Leiden University, Einsteinweg 55, 2300 RA, Leiden, the Netherlands*
[3]*Department of Chemistry and Hylleraas Centre for Quantum Molecular Sciences, University of Oslo, 0315, Oslo, Norway*
[4]*Zernike Institute of Advanced Materials, University of Groningen, Nijenborgh 4, 9747 AG, the Netherlands*
[5]*Max Planck Institute for Chemical Energy Conversion, Stiftstraße 34-36, 45470, Mülheim an der Ruhr, Germany*

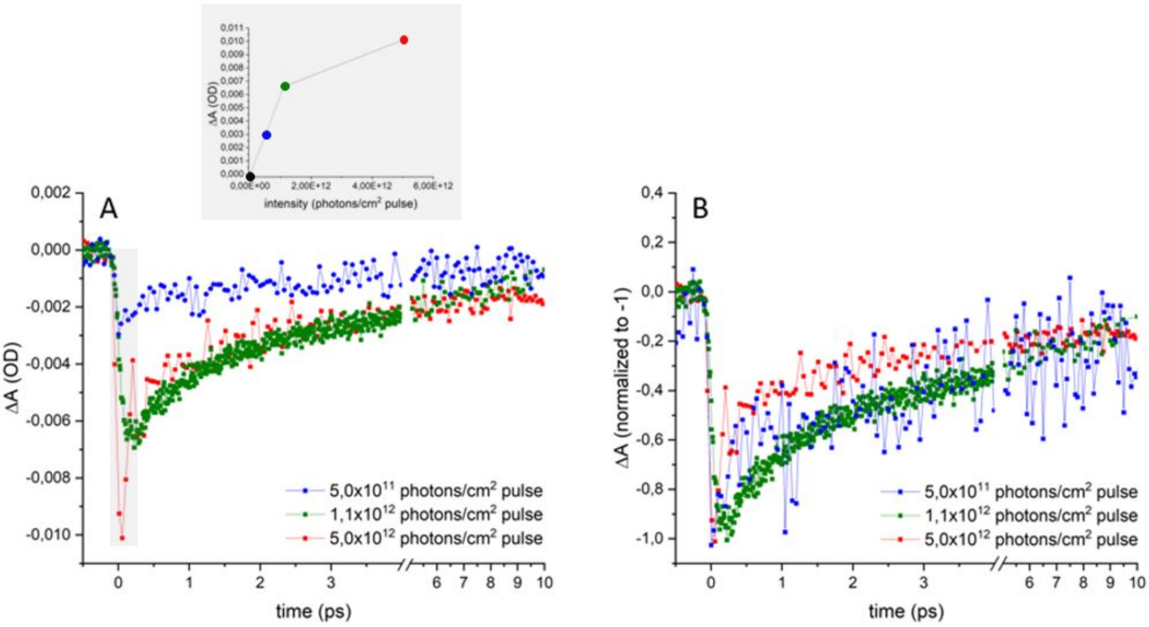

Figure S1. Intensity-dependent TA kinetic traces probed at 760 nm following 740 nm excitation of *Cba. tepidum* in de-aerated 50 mM pH 8.0 tris-HCl buffer at room temperature (A), showing that exciton-exciton annihilation is insignificant at the used intensity for measuring the TA data (1.0±0.1x10^{12} photons/cm^2 pulse) shown in the main text. At an intensity of ≥5.0x10^{12} photons/cm^2 pulse the decay normalized to -1 (B) becomes faster due to exciton-exciton annihilation, consistent with earlier work.[1] Also the maximum signal intensity around 100 fs does not increase linearly with the pump intensity any longer in this high intensity regime (inset A, red dot), in contrast to the low intensity regime (inset A, black, blue and green dots) where a linear relationship is observed.

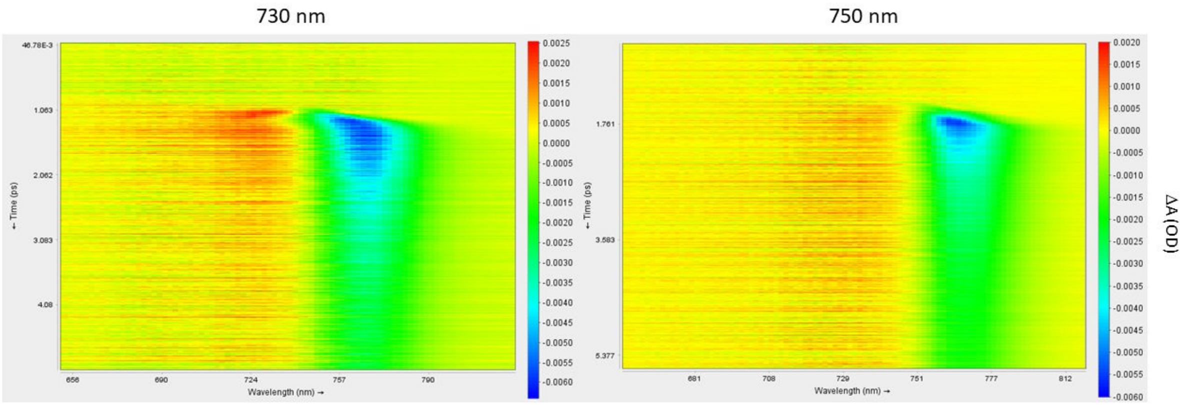

Figure S2. Early-time two-dimensional TA map of *Cba. tepidum* in de-aerated 50 mM pH 8.0 tris-HCl buffer at room temperature, showing the differential absorbance following 730 or 750 nm photoexcitation (with 1.0±0.1x10^{12} photons/cm^2 pulse) predominantly exciting the chlorosome.

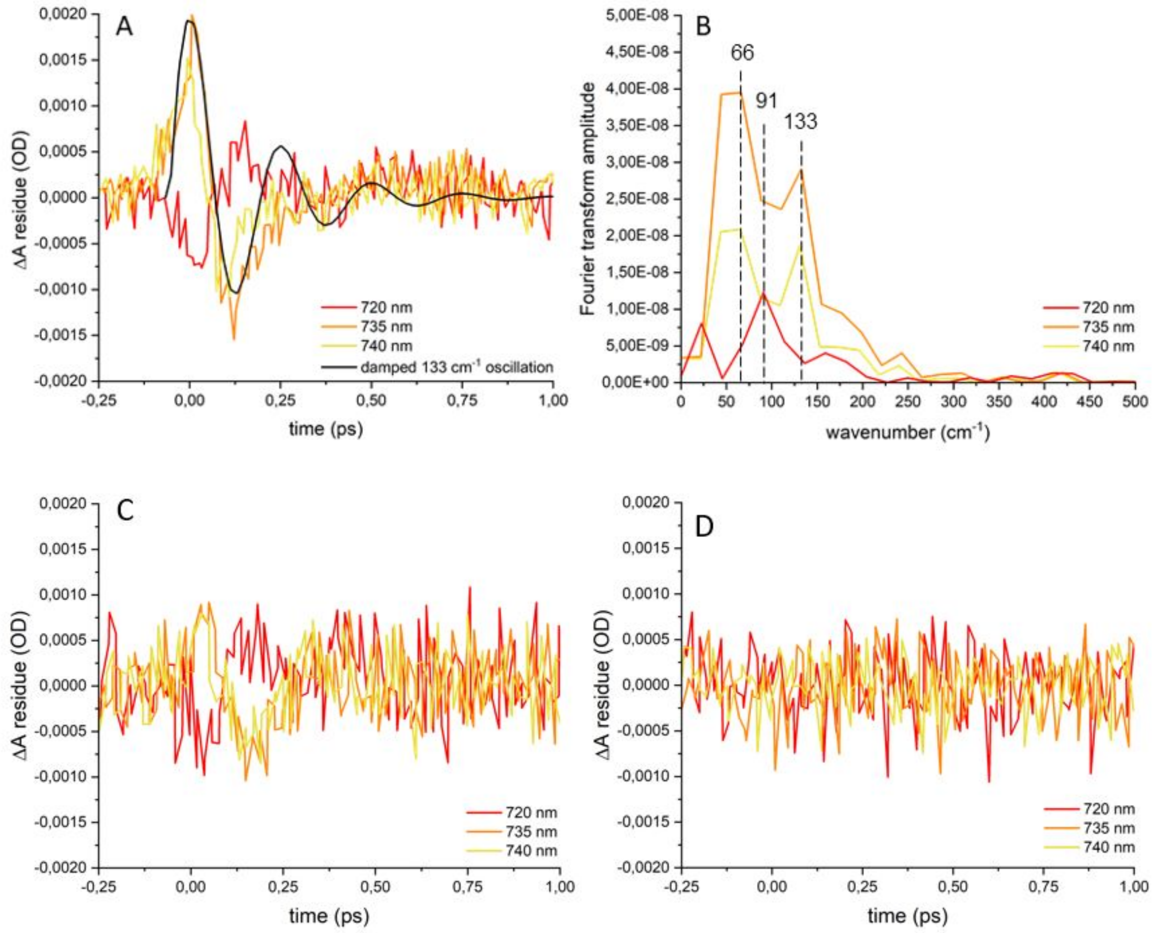

Figure S3. (A) Residuals of TA data and fits of the kinetic traces at 720, 735 and 740 nm following excitation with a center wavelength of 740 nm with 1.0±0.1x10^{12} photons/cm^2 pulse of *Cba. tepidum* in de-aerated 50 mM pH 8.0 tris-HCl buffer at room temperature, including a damped oscillation with a frequency corresponding to a wavenumber of 133 cm^{-1}. (B) Fourier transform of the residuals. Also included are the residuals at indicated probe wavelengths for excitation with a center wavelength of 730 nm (C) and 750 nm (D) with 1.0±0.1x10^{12} photons/cm^2 pulse.

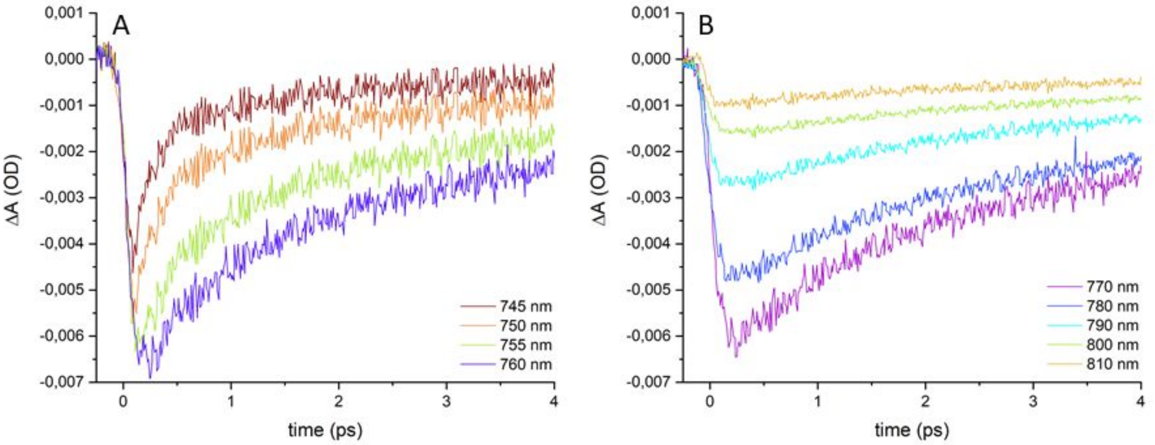

Figure S4. Early-time kinetic traces at various probe wavelengths following 740 nm excitation (with 1.0±0.1x10^{12} photons/cm^2 pulse) of *Cba. tepidum* in de-aerated 50 mM pH 8.0 tris-HCl buffer at room temperature. (A) The traces in the higher energy regime develop within the instrumental response time (IRT). (B) The signals in the lower energy regime develop both within the IRT and beyond. Furthermore, the decay becomes slower with lower probe photon energy, indicating downhill transfer in the chlorosome exciton manifold.

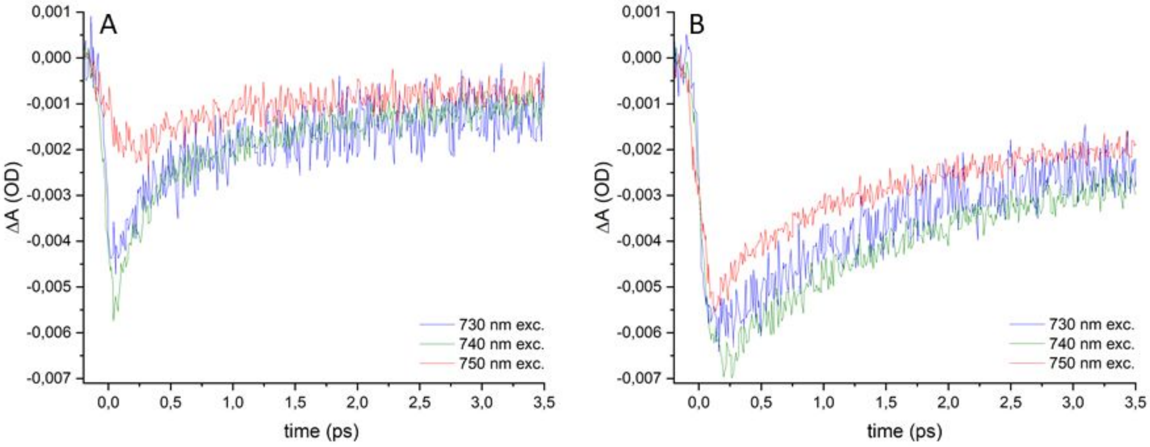

Figure S5. Kinetic traces probed at (A) 750 nm and (B) 765 nm of *Cba. tepidum* in de-aerated 50 mM pH 8.0 tris-HCl buffer at room temperature for 730, 740 and 750 nm excitation, with 1.0±0.1x10^{12} photons/cm^2 pulse. The larger differences in A relative to B indicate the following: 1) In addition to ground state bleaching (GSB), the TA signal at 750 nm is also due to stimulated emission (SE), which is more intense especially <1 ps for 730 and 740 nm excitation compared to 750 nm excitation. 2) The GSB/SE ratio at 765 nm is much less dependent on the photoexcitation wavelength, which can also explain the more similar dynamics in B compared to A.

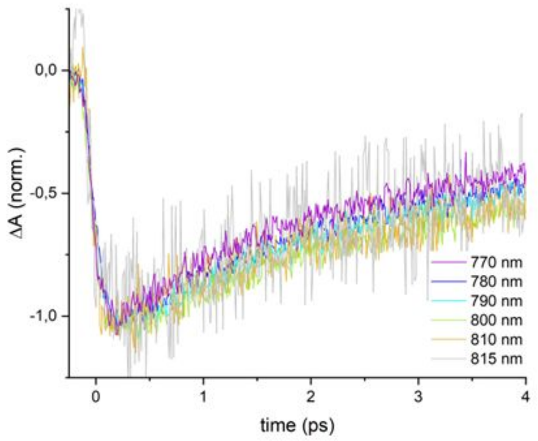

Figure S6. Early-time kinetic traces normalized to a maximum differential absorbance equal to 1 at the indicated probe wavelengths following 740 nm excitation with 1.0±0.1x10^{12} photons/cm^2 pulse; above *ca.* 780 nm the decay becomes independent of the probe wavelength.

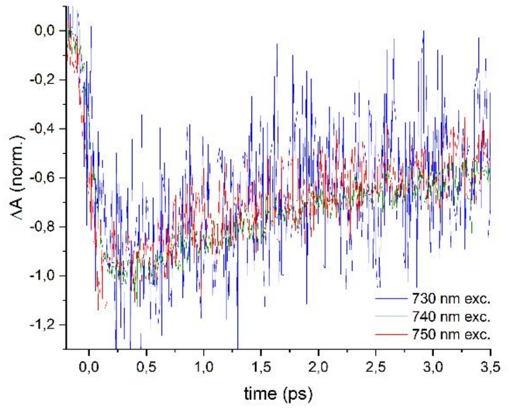

Figure S7. Kinetic traces at 805 nm normalized to an intensity of -1 of *Cba. tepidum* in de-aerated 50 mM pH 8.0 tris-HCl buffer at room temperature for 730, 740 or 750 nm excitation with 1.0±0.1x10^{12} photons/cm^2 pulse.

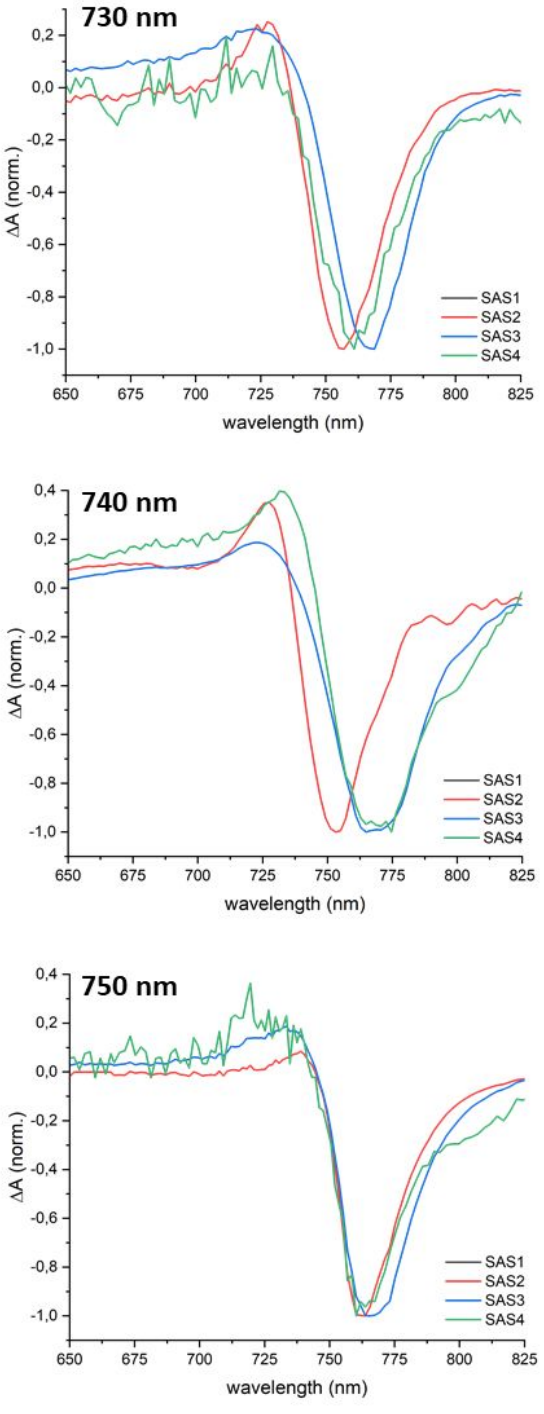

Figure S8. Species associated spectra (SAS) normalized to -1 from target analysis using the photophysical model shown in Figure 5 of the TA data recorded at 730, 740 and 750 nm excitation. SAS1-3 present the high, intermediate and low exciton manifold of the chlorosome. SAS1 needed to be forced to be to equal SAS2 to get a good fit. SAS4 presents the TA spectrum of the baseplate.

References

(1) Psencik, J.; Ma, Y. Z.; Arellano, J. B.; Hala, J.; Gillbro, T. Excitation energy transfer dynamics and excited-state structure in chlorosomes of Chlorobium phaeobacteroides. *Biophys J* **2003**, *84* (2), 1161-1179.
